# Application of Chemical Metallization of Photopolymer Structures Additive Technology in the Production of Components for Electronic Devices

**DOI:** 10.3390/mi14101897

**Published:** 2023-10-01

**Authors:** Mikhail D. Proyavin, Valentina E. Kotomina, Alexey A. Orlovskiy, Vladislav Yu. Zaslavsky, Mikhail V. Morozkin, Alexey V. Palitsin, Yuriy V. Rodin, Dmitriy I. Sobolev, Nikolay Y. Peskov

**Affiliations:** Institute of Applied Physics, Russian Academy of Sciences, 603950 Nizhny Novgorod, Russia; kotomina@ipfran.ru (V.E.K.); alexorlovskiy@ipfran.ru (A.A.O.); zas-vladislav@ipfran.ru (V.Y.Z.); morozkin@ipfran.ru (M.V.M.); pal@ipfran.ru (A.V.P.); rodin@ipfran.ru (Y.V.R.); sobolev@ipfran.ru (D.I.S.); peskov@appl.sci-nnov.ru (N.Y.P.)

**Keywords:** additive manufacturing technology, components for microwave devices, 3D-printing, copper plating, electron optics, electrodynamic structures

## Abstract

In this paper, we studied the operability of various components of vacuum electronic devices manufactured using the novel chemical metallization of photopolymer 3D-printed structures technology (CMPS), which is being applied at the Institute of Applied Physics, Russian Academy of Sciences (IAP RAS), for operation from microwave to sub-terahertz ranges. The key feature of this production method is the 3D printing (SLA/DLP, MJM technologies) of products and their further metallization. The paper presents the main stages of the process of chemical copper plating of polymer bases in various electrodynamic systems with complex shapes. A significant difference in the geometry and operating conditions of the created elements forms certain approaches to their production, as described in this work. Experimental studies of the implemented microwave components were carried out up to 700 GHz in the “cold” measurements; some electrodynamic structures were examined under conditions of sub-gigawatt peak power; and complex-shaped electrodes with cooling channels were tested under a continuous high thermal load. The conducted research has demonstrated the high potential of the developed methods of additive manufacturing of microwave device components and the prospects for their successful application in the described areas.

## 1. Introduction

Microwave radiation is currently being increasingly used in various fields of science and technology. Namely, large-scale work is underway to create controlled thermonuclear fusion reactors, where powerful microwave generators are actively applied [1,2,3]. On the basis of sources of millimeter and submillimeter radiation, promising complexes are being created for the treatment of cancer [4], the production of diamond films and disks by the chemical vapor deposition (CVD) method [5], and scientific installations for studying the properties of various media. Microwave devices are used to obtain nanopowders by the evaporation–condensation method [6], as well as compact ultraviolet sources [7], as a possible alternative to existing lithography facilities, etc.

In vacuum electronics, an important technology is associated with the creation of various electrodynamic components of complex shape that provide electron–wave interaction, transportation, and conversion of radiation, such as resonant cavities, waveguide elements, transmission lines, radiation input and output systems, mode converters, antenna systems, and others. The components of the electron-optical system are responsible for the efficient operation of the devices. The requirements for these elements are enhanced with the advancement into the higher frequencies up to the sub-terahertz and terahertz ranges.

The complexity of the shape of electrodynamic elements is characterized by inhomogeneities in the surface geometry, the size of which is comparable to the radiation wavelength λ. This imposes stringent requirements on the accuracy and surface quality of the manufactured component. It is generally accepted that the manufacturing inaccuracy of the component (i.e., deviation from the “ideal” design construction) and the roughness of its surface should be at least less than λ/10. Meanwhile, the generation efficiency of the devices and the radiation transportation are closely related to the conductive properties of the electrodynamic components, and therefore pure copper is used as the main material of their surface.

It should also be noted that in generators operating in long-pulse and up to continuous regimes (magnetrons, gyrotrons, etc.), a significant task is to ensure efficient cooling of the electron beam deposition region. This is caused by a large fraction of the residual energy in the electron beam after the electron–wave interaction (as for gyrotrons, the generation efficiency can reach values of about 50% at best, while the power of the electron beam in the continuous regime can be several MW). Accordingly, in addition to optimizing the current deposition process, it is necessary to use materials with high thermal conductivity and provide active cooling.

The continuous expansion of the field of application of microwave devices actualizes the process of creating new technologies for the production of its key components. High requirements for accuracy (at the micron level) in the manufacture of complex surfaces, including internal ones capable of operating in high vacuum and thermal loads, make the process of creating these devices extremely resource-intensive. In the field of microwave electronics, there is also a large share of scientific developments that require experimental prototypes to test theoretical ideas. The unreasonably high cost of prototyping often becomes an insurmountable challenge for small scientific teams, as well as the time frame and capabilities of the available machines. For many decades, CNC machines have been an indispensable tool for creating various metal elements with complex shapes. The precision of such machines is constantly increasing, as is their cost. However, the principle of operation remains the same: a workpiece of a standard shape is sharpened with special heads moving along different coordinates. The processing accuracy of advanced models is certainly impressive, amounting to fractions of a micron. However, such machines are rare and cost several million dollars. But even with such a machine, it is not possible to produce multiple components simultaneously. At the same time, the size of the milling head, as well as the drives, form restrictions on the minimum size of the inner surface that requires processing.

Currently, additive 3D printing technologies are being actively developed, which can potentially significantly facilitate the process of manufacturing elements for microwave electronics. 3D printing technologies can be globally divided into three parts: fused deposition modeling (FDM), stereolithography (SLA), and selective laser melting/sintering (SLM/SLS). The first, and the simplest one in terms of the principle of operation, is based on the layer-by-layer formation of a part by squeezing heated plastic through a nozzle. The presence of mechanical drives and motors for each of the axes, as well as the extrusion process dependent on the size of the nozzle and temperature effects, do not allow for achieving high product quality from the point of view of microwave electronics. SLA technology or its more advanced varieties, multi-jet printing/modeling (MJP/MJM), are much more suitable for these purposes. In contrast to FDM, the transverse pattern in the plane of the printing table (X and Y coordinates) is formed by a high-resolution LED lens array that is continuously improved. The characteristic pixel size is 15–20 microns, and there are no errors caused by the mechanical movement of the print heads. In these printers, the mechanical part is located only on the vertical *Z*-axis, which is responsible only for the height of the layer and is not subject to frequent and rapid movements and, therefore, the occurrence of unwanted vibrations. However, despite the high quality of products, this technology is fundamentally unable to print the metal products required for microwave technology. One solution to this problem may be to cover the working surface with a conductive layer. The most common option is coating with special ink or silver paint with a further coating of copper by electroplating [8,9,10] (). This approach is suitable for creating a certain class of low-frequency microwave electronics components but does not seem promising due to the difficulties of applying a uniform layer to complex surfaces of elements. In addition, this sublayer is a problem for creating all-copper elements when removing the printed mandrel. Printing with both plastic and metal is possible on SLM/SLS printers. The principle of operation is based on layer-by-layer sintering of nanopowder under the action of a laser. Each layer, as in previous technologies, is a specific cross-sectional pattern of the model. However, when it is formed due to thermal effects, nearby nanopowder particles “stick” to the main structure of the pattern. This process results in an extremely rough surface unsuitable for high-frequency applications (tens of GHz and above) [10]. Without additional surface treatment, roughness can range from tens to hundreds of microns. At the same time, in the case of SLM technology, there is a significant problem of printing products with internal cavities and overhanging elements. For their correct formation, special supports are required, which must be removed in the next step. Obviously, this significantly complicates the whole process, especially in comparison with the same CNC machines. This problem is much less manifested in the SLS technology because, in this case, the nanopowder is a support itself. However, in the case of SLS, powder particles are only soldered to each other without forming a monolithic structure, unlike SLM. The result is a porous structure that is not suitable for high vacuum operation.

All three 3D printing technologies are not suitable for efficient use in the field of microwave electronics. However, their advantages over CNC machines are extremely attractive. The presence of a method for fast, cheap, and high-precision (for a certain class) creation of components of electronic devices obviously provides significantly more opportunities to test theoretical ideas using experimental methods. In some cases, it is possible to create elements with complex surface geometry using additive technologies. Meanwhile, such tasks are difficult or completely impossible to implement using standard methods. The main disadvantage of photopolymer printing technology is the impossibility of obtaining metal elements, which can be solved by additional processes of metallization of the dielectric mandrel. This paper describes the process of developing CMPS technology [11], as well as the adaptation of this technology to the production of copper components for electronic devices [12], operating in various frequency ranges, thermal loads, high vacuum conditions, and intense electromagnetic fields.

## 2. Chemical Aspects of CMPS Technology

To create elements with a thin copper layer, the polymer base had to have high strength and a minimum coefficient of thermal expansion. For these purposes, Gorky liquid force resin was chosen [13]. In the case of the implementation of all-copper components, Gorky liquid castable photopolymer resin was used as a material for the mandrel. This resin is distinguished by the fact that under certain heating conditions (up to 650 °C), it can be completely burned out. However, with the simple burning of a monolithic photopolymer mandrel, already at a temperature of about 200 °C, due to the difference in the coefficients of thermal expansion, (CTE) of the metal and the photopolymer, deformation and destruction of the metal housing of the microwave component occur. Therefore, the procedure for creating a model and removing the mandrel follows a certain technological process. The main characteristics of the resins used are given in Table 1.

Various approaches have been developed for the metallization of plastics, including magnetron sputtering, galvanic, and chemical methods, along with a combination of these methods. In general, it is necessary that the applied metal coating have a sufficiently strong adhesion to the surface to be coated. In turn, the surface must have certain physical and mechanical properties that determine its joint use with the coating and have chemical properties that would allow it to be easily processed in the right solutions and/or on the necessary equipment.

For open surfaces, magnetron sputtering is practically an ideal option for coating dielectrics [14,15]. However, it does not allow metallization of parts with complex external and, especially, internal surfaces. Galvanic methods are convenient for building up an unlimitedly thick layer of metal, but they involve the implementation of primary metallization by chemical means to create an electrically conductive layer.

Coating a surface of almost any shape with a relatively thin layer of metal is possible by chemical (non-galvanic) metallization, although the specific amount of deposited metal is determined by the presence of fresh reagents in this place and can differ markedly from point to point.

The processes of chemical and galvanic metallization are well studied and successfully applied in industry. However, it has been worked out only for certain types of polymers. In general, these methods include the following main stages of preparing the plastic surface for primary metallization:

- Cleaning step, including at least degreasing in alkaline solutions or organic solvents. The choice of solution composition and equipment is made depending on the type, degree of surface contamination, and nature of the plastic. Meanwhile, such compositions and processing regimes are selected in which fatty contaminants are easily removed and the processed material does not dissolve, swell, or crack.
- The etching stage is a chemical process that changes the structure and chemical properties of the plastic surface. At the same time, it is given the required roughness, hydrophilicity, and reactivity during subsequent coating operations. The etching step may be preceded by a conditioning (pre-etching) process. Pre-etching includes treating the plastic surface with organic solvents, their mixtures or emulsions, solutions of acids, alkalis, and salts, as well as heat treatment, irradiation, sonication, and other types of exposure. Such processes remove or loosen the surface layer, helping to improve the etchability of the dielectric. At the same time, the duration and temperature of the etching are reduced, and the period of operation of the solution is extended.
- Stage of surface sensitization: treatment of the plastic surface with a reducing agent solution, which in most cases is used as hydrochloric acid solutions of tin (II) chloride; the Sn^2^ ions contained in them, which are in solution, are sorbed by the plastic surface and undergo further hydrolysis with the formation of poorly soluble products that tightly fit on the treated surface.
- Stage of surface activation: treatment of the sensitized surface with solutions of compounds of catalytically active metals. A solution of one of the noble metals (palladium, silver, gold, platinum, etc.) can be used as an activator. As a result of activation, Sn^2+^ ions are replaced on the plastic surface by catalytically active metal ions.

As a result of all this preliminary preparation, chemically active centers of noble metals embedded in the plastic surface are created, which are subsequently replaced by ions of the required metal during the autocatalytic process of primary chemical metallization of the polymer surface. The further metallization process, depending on the required characteristics of the product being created, can be carried out by building up the metal layer by chemical or galvanic means.

US Patent 10,563,308 [16], which describes a process for obtaining a three-dimensional structure having a horizontal and/or vertical resolution of elements in the range from several hundred nanometers to several hundred microns. Such structures have at least one metal deposited on them, including dissolving one salt metal in a resin containing one or more photopolymers; curing parts of the resin to form a three-dimensional structure; and using metal ions included in the surface of the three-dimensional structure as catalysts for chemically depositing at least one metal directly on the surface of the three-dimensional structure. Thus, this patent mentions the possibility of carrying out the metallization of a photopolymer part by bypassing the stages of sensitization and activation.

The advantage of this technology is the possibility, by using masks and precursor materials, to create areas on the surface of the fabricated structure containing activation centers of various metals for further metallization. There is also a chance for the metal coating of photopolymers, which initially do not lend themselves to primary chemical metallization at all. However, such a process seems to be very costly and time-consuming since it is necessary to repeatedly change precursor materials in the tank with an admixture of ions of each specific metal. At the same time, it is completely unclear how to control the final concentration and distribution of the resulting activation centers for further chemical metallization on the surface of the structure, as well as the hydrophilic and adhesive properties of this surface, which are so necessary for high-quality primary metallization.

In addition, in the specified analog, there is no information about the stage of cleaning the surface of the structure before metallization. At the same time, it should be taken into account that, unlike products made from conventional plastics, the surface of products made from photopolymers produced by 3D printing is initially absolutely not suitable for further metallization since it is covered with a sticky, uncured resin. The presence of fragments of such an underpolymerized resin does not allow preparing the surface of the product for further metallization, even in the case of an ideally selected reagent.

An obvious solution to this problem is the ability to illuminate the finished product after printing in a special camera with LEDs with the required wavelength of light. However, this path is not always suitable. First, if there are many internal channels in the structure, then the illumination is extremely inefficient. Secondly, if the quality of the surface is very important, additional light can lead to a deterioration in the mechanical properties of the plastic due to the “aging” of the upper layers of the photopolymer due to improperly selected thermal and photocuring modes. That is why, during the metallization of a mandrel made of a photopolymer, the features of the procedure for cleaning and preparing its surface are of primary importance.

The manufacture of microwave components of complex shapes with a developed metal working surface and high accuracy and quality is carried out by the method detailed in the patent [8]. By 3D printing, a mandrel is made of a photopolymer with a determinedly complex surface. The surface of the photopolymer mandrel is subjected to chemical liquid treatment in order to degrease it and remove underpolymerized residues of the photopolymer resin from its surface. First, degreasing is carried out with a solution of the following composition: Na_3_PO_4_, Na_2_CO_3_, KOH, liquid glass, H_2_O. Depending on the type of polymer used, the temperature of the composition is maintained in the range of 50 ± 10 °C. The procedure is performed in an ultrasonic bath. The commonly used standard processes for cleaning and degreasing plastics in isopropyl alcohol lead to a noticeable departure in the geometric parameters of the mandrel (alcohol strongly eats into the plastic structure, which swells and deforms due to this), which is unacceptable for its further use as components of high-frequency electronics. This process is carried out several times, followed by washing the mandrel with distilled water, which removes traces of the degreasing solution. The number of cycles depends on the complexity of the mandrel geometry and the presence of hidden cavities and internal channels. Finally, the mandrel is dried with compressed air.

Then, the surface of the mandrel is micro-etched from a photopolymer, which provides better adhesion to the metal layer. The chemical composition of this process is based on the widely used chromic solution (concentrated sulfuric acid and chromium (VI) oxide). The oxidizing abilities of potassium dichromate are based on the oxidizing capabilities of sulfuric acid and its effect on organic macromolecules on the surface of a photopolymer mandrel. The composition of potassium dichromate is chosen in such a way that the impact of sulfuric acid, with its pronounced oxidizing abilities, directly on the photopolymer mandrel is minimized.

In our case, the goal is to create microroughnesses at the nanometer level, which subsequently improve the adhesion of the deposited metals to the surface of the photopolymer mandrel. In addition, the strong oxidizing properties of the specified solution allow for additional cleaning of the mandrel surface. K_2_Cr_2_O_7_, which is usually used for these purposes, has a worse oxidizing ability and does not allow effective removal of underpolymerized products from the surface of the mandrel, but it is more aggressive and “burns” the surface more strongly. The processing time in the solution is minimal (about 15 min) to prevent “burning” the surface. The operation of micro-etching is carried out at a temperature not exceeding 25–30 °C, while an increase in temperature affects the rate of the oxidation reaction of macromolecules on the surface of the photopolymer mandrel. As a result, highly etched areas are formed, which leads to a deterioration in the quality of the surface.

After treatment of the photopolymer mandrel in an oxidizing solution, its surface should be washed in distilled water. A series of washes varies from 2 to 5, depending on the complexity of the treated surface. The first flush is carried out in a chromium-ion trap.

The surface of the photopolymer mandrel cannot be exposed to a more concentrated solution of potassium dichromate. Also, insufficient cleaning of its surface from residual products of the polymerization reaction is possible. For the purpose of final cleaning and additional micro-etching of the surface of the photopolymer mandrel after chemical etching in a weak solution of potassium dichromate, an additional oxidizing treatment of its surface is carried out in an ammonium peroxide solution. Treatment with oxidizing agents is performed at a temperature of 40–50 °C.

In a warm solution, atomic oxygen, as a product of the decomposition of hydrogen peroxide, oxidizes the residual underpolymerized resin macromolecules, additionally “drying” the surface of the mandrel. This solution in our case can be used only at the final stage of cleaning, since the use of this oxidizing solution alone does not provide a deep and complete removal of organic residues from the polymerization reaction from the plastic surface. Processing time varies within 5–10 min.

The disadvantage of this procedure is the imparting of hydrophobic properties to the treated surface, which is unacceptable for further sensitization and activation procedures. Therefore, isopropyl alcohol is used to transfer the surface from the hydrophobic to the hydrophilic state, a treatment that does not exceed 10 min at a temperature of 40–50 °C. Here, isopropyl alcohol is used not as an additional degreasing agent but as a wetting agent. This allows you to give the treated surface the desired hydrophilic properties that were lost due to previous chemical treatments. Thus, when preparing the mandrel surface for sensitization and activation in the case of photopolymers, the processes of cleaning and etching are practically combined.

Further stages of sensitization and activation in preparing the surface of a photopolymer mandrel for primary metallization are fairly standard. The mandrel is processed in a reducing agent solution. A hydrochloric acid solution of tin (II) chloride is universal. The sensitization process is carried out in fresh solutions of tin dichloride with exposure for 5–10 min, after which the mandrel made of photopolymer is washed in several portions of distilled water to trap hydrolysis products from its surface.

For a more complete trapping of excess Sn^2+^ ions from the surface, additional exposure is carried out in a 2% NaOH solution. Next, activation is carried out—substitution from the surface of Sn^2+^ ions for ions of the catalytically active metal Ag^2+^ from a solution of silver nitrate. Then, the mandrel made of photopolymer is washed in several portions of distilled water.

The further autocatalytic process of primary chemical metallization of the surface of a photopolymer mandrel is considered using copper as an example since, in 90% of cases, this metal is used to manufacture microwave components. This is explained by a number of properties valuable for microwave electronics: copper provides low signal losses, can be easily galvanized, allows efficient heat removal, etc.

After the surface of the photopolymer mandrel is activated, chemically active centers are created on it by the chemical substitution of one metal with a more active one. For further deposition of metallic copper, the primary deposition of copper is carried out in a saturated solution of the following composition: CuSO_4_ × 5H_2_O, NiCl_2_ × 2H_2_O, Na_2_CO_3_, NaOH, Trilon B, K_3_[Fe(CN)_6_], KNCS, and formaldehyde (very low concentration, dangerous carcinogen).

The process of primary metallization is often accompanied by the formation of local delaminations of the metal under the coating. This defect is most noticeable on incompletely cleaned surfaces, which makes it possible to determine local “dirty” zones and carry out additional cleaning in chemical solutions after removing the copper coating. Several cycles of this procedure allow you to achieve a smooth surface. In addition, the appearance of bubbles-delaminations can also be associated with the formation of gas in the course of a chemical reaction during metal deposition. Properly selected quantitative concentrations of formalin, which start the process of autocatalytic copper deposition, make it possible to achieve a moderate release of gaseous hydrogen and slow down the reaction, which can significantly reduce the number of microswellings since the gas has time to escape before the metal film is tightened.

The time of primary metallization depends on the degree of complexity of the surface of the photopolymer mandrel, the quality of the previous chemical treatment operations, and the processes of microetching the surface of the mandrel—the so-called primary surface roughness. In our case, the exposure time in the chemical copper plating solution is 30–40 min. This is quite enough to create a dense, conductive primary metal layer of copper. Next, the copper layer is grown to the desired thickness by electrochemical (galvanic) deposition in a solution of the composition CuSO_4_ × 5H_2_O, H_2_SO_4_, C_2_H_5_OH, and shine additive.

In a particular case, before the sensitization process at a low level of adhesion of a particular photopolymer, plasma-chemical treatment of the surface of the mandrel from this photopolymer is additionally carried out. Processing is performed by low-temperature plasma, which, along with the surface cleaning of the mandrel from residual underpolymerized areas of the photopolymer that have undergone liquid chemical treatment, also changes the properties of the surface layer. Modification of the near-surface layers of the mandrel occurs due to the breaking of macromolecule bonds and the formation of polar groups. In addition to improving the adhesive properties of the photopolymer, in this case, there is an improvement in the wettability of the surface of the mandrel, which can be seen during the subsequent chemical metallization process. The rate of metal deposition from the saturated solution increases, and the metal deposition proceeds evenly over the entire plasma-treated surface of the photopolymer mandrel.

## 3. Adaptation of CMPS Technology to the Production of Various Types of Microwave Components

### 3.1. Microwave Elements for “Cold” Measurements

The basic stage in the creation of microwave components using the technology under consideration is the coating of a 3D-printed workpiece with a thin layer of copper (about 10 μm). This process eliminates the long-term growth of a thick metal layer by electroplating. Accordingly, for applications where the microwave components do not have mainly thermal loads, this method allows the production of parts at high speed. A large class of tasks in the scientific and industrial spheres is to check the performance of various waveguide elements on a vector network analyzer (VNA), the signal of which is several milliwatts. This process allows evaluation of the efficiency of the device by examining the so-called S-parameters (reflection and transmission coefficients) in various frequency ranges. In microwave components in such experiments, the specific structure of the RF field is considered. To excite it, unique mode converters are needed that form the required type of oscillations from the standard modes of VNA waveguides. The creation of such electrodynamic systems for each specific task is also a promising and important area of application of CMPS technology.

Operation in high-frequency ranges (in the conducted experiments, the operating frequency reached 700 GHz) imposes stringent requirements on manufacturing accuracy and the quality of the copper coating. First of all, in order to obtain the required dimensions with high accuracy, it is essential to control the shrinkage coefficient of the photopolymer resin. It is advisable to adjust the model after its preliminary printing and make measurements on each of the planes. Considering the wavelength range up to 500 µm, it is important to take into account the requirements for errors better than λ/10 and sharpness about λ/100. To meet these requirements, the printing processes, cleaning processes, and surface metallization processes have been optimized.

**Figure 1:**
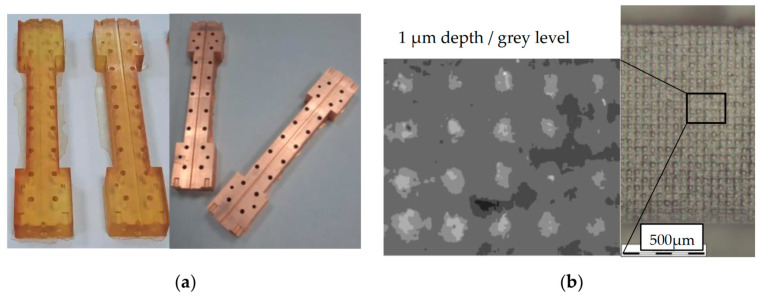
(**a**) Photo of printed parts of a rectangular waveguide with a cross section of 1.65 × 0.83 mm^2^ (WR7) before metallization (left) and after it (right); (**b**) Photo of the surface structure of the waveguide channel under a microscope with an analysis of the amplitude of the corrugation caused by the pixel structure of the 3D printer matrix.

We will most clearly demonstrate the process of creating an electrodynamic element on waveguides consisting of two parts. The simplest example is a regular rectangular waveguide, which is used in the frequency range up to 170 GHz. The printed halves of the waveguide are shown in Figure 1a. Then, after cleaning and surface preparation procedures, a copper layer with a thickness of about 5 microns is chemically grown.

**Figure 2:**
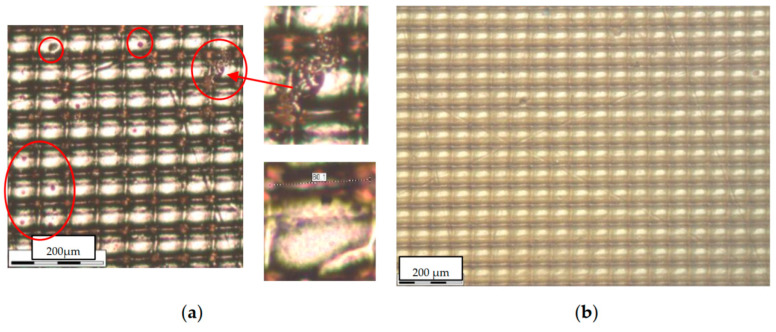
(**a**) Microscope image of the structure of the sector of the copper-clad waveguide surface with the presence of foreign inclusions that degrade the quality of the surface; (**b**) photo of a clean surface obtained by optimizing the cleaning and plating process.

The presence of the element in the bath of chemical copper plating is limited in time. This is due to the active release of gases, which can cause the formation of microbubbles on the original surface. Therefore, after the element is completely covered with copper, it is placed in a galvanic bath, where the copper layer grows up to 10–15 microns. In the case of properly selected cleaning and copper plating conditions, it is possible to achieve a surface quality that completely replicates the original polymer blank. Therefore, the quality of the product entirely depends on printing. In this case, for current needs, we use a Phrozen 8K mini [17] SLA printer with a pixel size of about 20 microns for tine elements and a Phrozen 8K MEGA (pixel size of about 50 microns) for the bigger ones. Observing through a microscope the periodic structure of the waveguide surface (caused by the resolution of the matrix) (Figure 1b and Figure 2a,b), the exposure times were selected so as to minimize the depth of the dip between adjacent hillocks. In the case of optimal exposure time, the depth was reduced to about 3–4 µm (Figure 1b). After printing, it is extremely important to prevent dirt (fibers, dust, etc. in Figure 2a) from getting on the surface to make it clear (Figure 2b).

**Figure 3:**
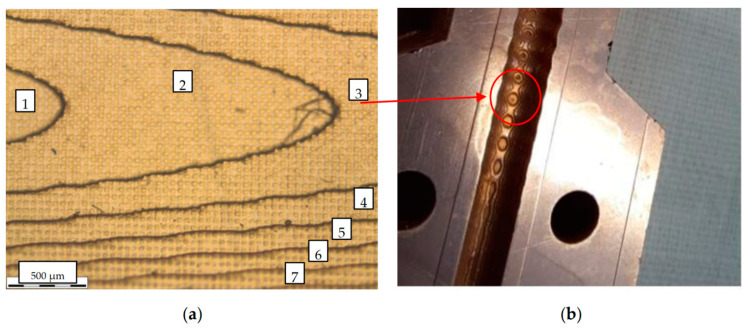
(**a**) Microscope image of the structure of a copper-plated printed surface, which has a small angle of inclination of the surface relative to the horizontal plane of printing. The numbers 1–7 show the printed layers characterizing the depth/height of the surface; (**b**) a photo of this metalized part and its analyzed area of the inner surface of the waveguide.

In the case of more complex waveguides, for example, with a variable cross section of the internal channel, it is important to choose the correct location of the part on the printing table. For example, in the case of a smooth change in the surface profile in the horizontal printing plane (Figure 3b), this geometry will be represented as steps formed by printing layers along the vertical axis (see Figure 3a). The decrease in layer thickness is currently limited to a value of about 10 μm. Nevertheless, this layer height will be quite noticeable at small angles of profile change at relatively large lengths. In such cases, the part should be placed vertically.

The first results of cold measurements of microwave components created using CMPS technology have been published in [18]. Successful tests of the obtained electrodynamic structures imply further development in higher frequency ranges. Preliminary studies of coatings on various microwave devices have shown that the difference from ideal copper is 5–20%, depending on the complexity of the geometry. Taking into account the extremely high conductivity of copper, this difference can be considered small enough to create microwave components in the frequency ranges above 170 GHz.

**Figure 4:**
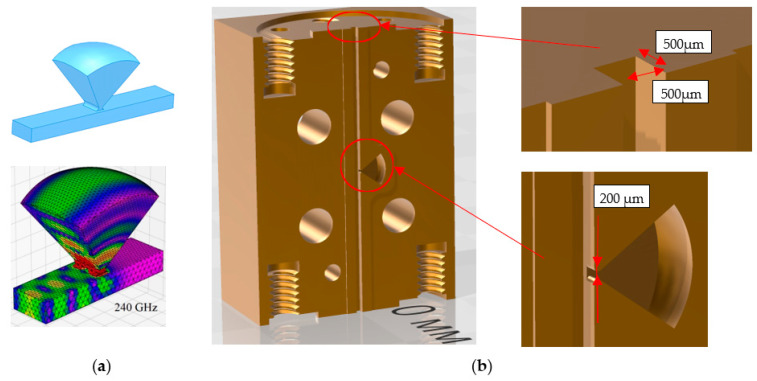
(**a**) View of the inner surface of the waveguide and the resonator of the notch filter (top) and a map of the distribution of the RF field at the resonant frequency in the working space (bottom); (**b**) 3D model of one of the notch filter halves. Additionally, the resonator and its coupling hole with the waveguide are shown, as well as the profile of the contact surface of the two parts: the height of the profile rise is 100 μm the width is 1 mm.

As the first stage of development of the CMPS technology for the frequency range of 200–300 GHz, manufacturing a waveguide notch filter with a center frequency of the stopband of 240 GHz [19] was carried out. A distinctive feature of this filter is the pyramidal shape of the resonator. It is possible to obtain selective excitation of only one eigenmode of the oversized resonator in the frequency range from 200 GHz to 285 GHz by choosing the location of the coupling hole on one of the faces of the truncated pyramid, the opening angles of the pyramid, and the shape of the upper face (Figure 4a). The 3D model of the device is shown in Figure 4b.

**Figure 5:**
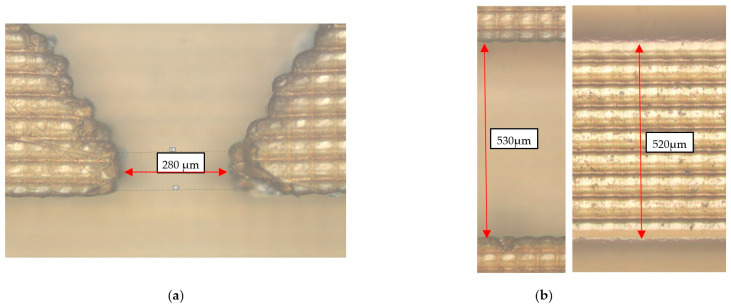
Microscope image of the surface structure and key dimensions of the copper-free notch filter in the region of the resonator, coupling hole (**a**), and waveguide (**b**). The focus of the microscope objective is directed to the ends of the side walls of the resonator (**a**) and waveguide (**b**) (left). The focus of the microscope objective is directed to the bottom of the waveguide (**b**) (right).

It should be noted that the manufacture of this filter by mechanical processing is a very complex technological task. On the contrary, its manufacture is not difficult due to the proposed additive technology. The main task in this case is to ensure a sufficiently low surface roughness and to maintain the dimensions of the resonator and its coupling with the waveguide. The latter problem was solved by printing several versions of the waveguide, differing in the size of the waveguide elements both in the smaller and larger directions. Also, the shrinkage of the polymer (about 0.3%) and the thickness of the copper coating (30 µm per wall) were considered in the model. At the time of the creation of this microwave component, a Phrozen 4K mini printer was used with a characteristic pixel size of about 40 microns. At the same time, due to the optimization of illumination, the depth of the periodic pixel structure on the surface of the element was about 4 μm. The final ordered roughness of the structure is shown in Figure 5. It also demonstrates the actual dimensions of the main sections of the element (coupling with the resonator (Figure 5a) and waveguide (Figure 5b) up to the copper coating). The difference from the specified dimensions of the metallized sample is on average about 30 µm (15 µm per wall), which gives an error of about 5%. To minimize it, a notch filter version with reduced print sizes was chosen (smaller by 10 μm per wall).

**Figure 6:**
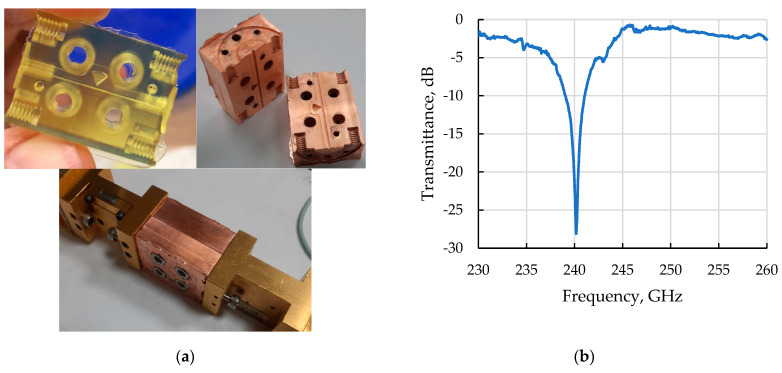
(**a**) Photograph of printed and copper-plated notch filter; (**b**) The results of measuring the transmission coefficient through the notch filter on a vector panorama.

A photograph of this notch filter and its connection to a VNA is shown in Figure 6a. During the experiment, the radiation transmission coefficient was measured in the range of 230–260 GHz. The measurement results are shown in Figure 6b. It can be seen that, with good accuracy, the notch frequency is in the calculated value of 240 GHz with a suppression value of about −30 dB. However, despite the successful operation of the device and the high performance of the notch filter, the calculated value of the suppression is about −50 dB. This discrepancy may be due to several factors. Firstly, the calculations were carried out in the approximation of an ideal conductor. Secondly, the correct alignment of the two halves of the filter can play an important role, especially in the region of the resonator and the coupling hole.

**Figure 7:**
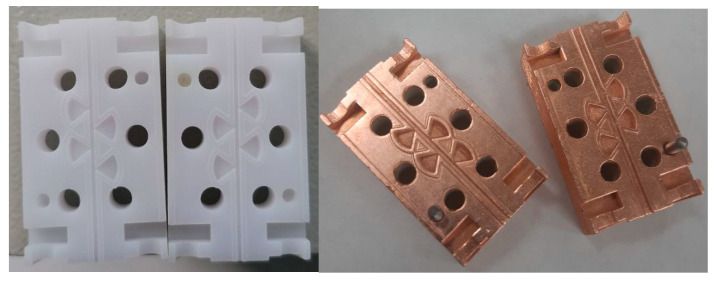
Photo of a printed four-cavity notch filter before copper coating (**left**) and after (**right**).

The demonstrated capabilities of the technology allow for further modernization of these notch filters, which have improved signal suppression parameters. At present, a fabricated version with four resonators is being considered and tested (see Figure 7). However, in this case, the issues of mutual adjustment of the two halves to ensure resonance at one frequency come to the fore.

Further advancement into the submillimeter range took place with the example of the creation of 0.7 THz advanced Bragg structures of cylindrical geometry based on the coupling of propagating and quasi-cutoff waves. These structures are designed for the project of a high-power sub-THz/THz band long-pulse free-electron laser (FEL) being developed based on the linear induction accelerator LIU (5–10 MeV/1–2 kA/200 ns) at the Budker Institute of Nuclear Physics (BINP RAS, Novosibirsk, Russia) in collaboration with the IAP RAS [20]. One of the key problems in the implementation of such an FEL is the development of an electrodynamic system capable of providing a stable single-mode operating regime for narrow-band generation under conditions of substantial oversize. To date, the Bragg resonators proposed in [21] in the form of waveguide segments with shallow corrugation of the side walls have been widely used as electrodynamic systems of relativistic masers in the microwave band. However, an increase in the transverse dimensions of the conventional Bragg resonators (based on the coupling of two counter-propagating paraxial waves) is associated with a loss of selectivity over the transverse mode indexes. At the same time, the advancement of powerful FEL oscillators into the sub-THz range inevitably requires an increase in the oversize parameter (i.e., the size of the interaction space to the wavelength ratio). On the one hand, this is necessary to form a channel for transporting an intense electron beam, but on the other hand, it is also essential to reduce Ohmic losses. A promising approach to solving this problem can be considered the use of the so-called advanced Bragg resonators, the distinctive feature of which is the coupling between the propagating and quasi-cutoff waves [22]. To implement the indicated feedback mechanism, the corrugation period should be approximately twice as long as in conventional structures. The use of quasi-cutoff waves in the advanced Bragg structures leads to a significant rarefaction of the eigenmode spectrum and an improvement in their selective properties compared to conventional analogues. According to the modeling performed both on the basis of averaged models and the FDTD method, the advanced cylindrical Bragg structures make it possible to provide selective excitation of the operating mode at a diameter up to Ø ~ 50λ at the sub-THz frequency range, which seems to be sufficient for the formation of a channel for transporting intense relativistic electron beams.

**Figure 8:**
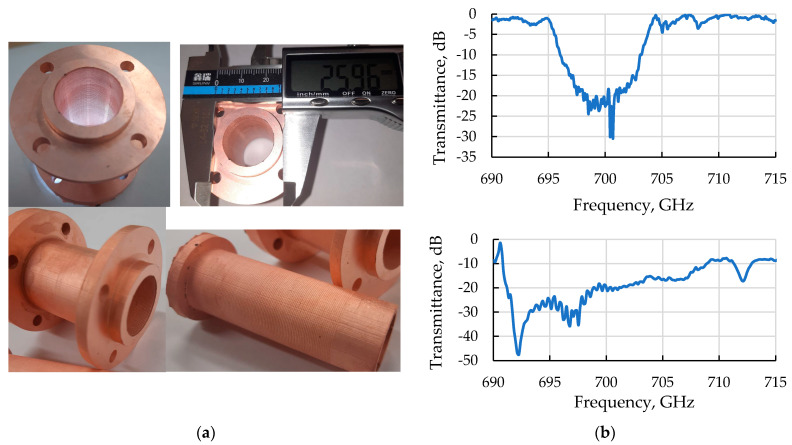
(**a**) Photographs of advanced Bragg structures of cylindrical geometry created using the CMPS technology, including the negative form of the model for further building up a thick layer and removing the polymer (bottom right); (**b**) The results of the 3D CST simulations of the transmission coefficient through the structure (top) and results of its “cold” tests (bottom).

The proposed Bragg structure, elaborated and implemented by 3D printing technology to operate at a frequency of 0.7 THz, has a diameter of 20 mm, a length of 50 mm, a corrugation period of 0.43 mm, and a depth of 0.15 mm. These parameters provide effective coupling and mutual scattering of the paraxial TE_1,1_ and the quasi-cutoff TE_1,45_ waveguide modes. According to the 3D CST simulations, the designed structure can selectively reflect the FEL operating wave of the TE_1,1_-type into the backward wave of the same TE_1,1_-type (via excitation of the mentioned high-order cut-off wave) with about 90% power efficiency. A view of the 3D model and a photograph of its implementation using CMPS technology are shown in Figure 8a.

To measure the properties of the structure at a low power level (the so-called “cold” tests), symmetrical quasi-optical converters were developed and manufactured. The single-mode waveguide output of the vector network analyzer was connected to a sufficiently long cone-shaped horn, which produces the incident wave-beam of the TE_1,1_-type at the input of the tested advanced Bragg structure. A similar scheme was used at the output of the structure when measuring the transmission coefficient. The transmission coefficient measurements in the described “cold” tests are in good agreement with the theoretical predictions (see Figure 8b).

### 3.2. Microwave Components for High-Power Applications

#### 3.2.1. High-Power Pulsed Applications

In most microwave devices, the operating regime is associated with the presence of high power from both radiation and its source, an electron beam. In currently implemented microwave generators, the radiation power reaches gigawatt-level in the pulse regime and in continuous mode (megawatt level). In such devices, first of all, it is essential to solve the problem of the deposition of high-energy particles on the device walls. To perform the correct operation, it is necessary to provide not only efficient heat removal from the vacuum volume but also to prevent deterioration of the vacuum. For these purposes, the thickness of the copper layer should be more than 1 mm. For example, in the case of a thin coating, even a short pulse of a powerful electron beam can destroy the copper layer and contaminate the vacuum volume with the evaporated photopolymer.

To create electrodynamic and electron-optical structures with a complex surface, further galvanic build-up of the copper layer is required. This process is very rough; it is almost impossible to achieve a high-quality operating surface due to the appearance of dendrites and the inhomogeneous build-up of copper on complex geometry. Therefore, to create such products, an inverse form of the model is printed, on which a copper layer is subsequently created. This allows for the maintenance of a high-quality operating surface while at the same time building up a thick layer of copper galvanically. As a final step in creating the model, the resin mandrel is removed.

This direction of CMPS technology has been optimized for the creation of powerful spatially extended Cherenkov-type oscillators based on a novel type of slow-wave structures (SWS)—the 2D-periodic SWS, which are being developed at the IAP RAS [21,22]. In the process of joint work, more than ten structures of complex shapes were successfully created and are currently being tested in the frequency range of 32 GHz to 150 GHz. Using these structures in this experiment, a record for microwave pulse generators output power level of up to 200–250 MW in the Ka-band (corrugation period *d* ≈ 7.7 mm, oversize factor Ø/λ~5) [23] and 120–150 MW in the W-band (*d* ≈ 3.6 mm, Ø/λ ~ 10) [24] was achieved.

**Figure 9:**
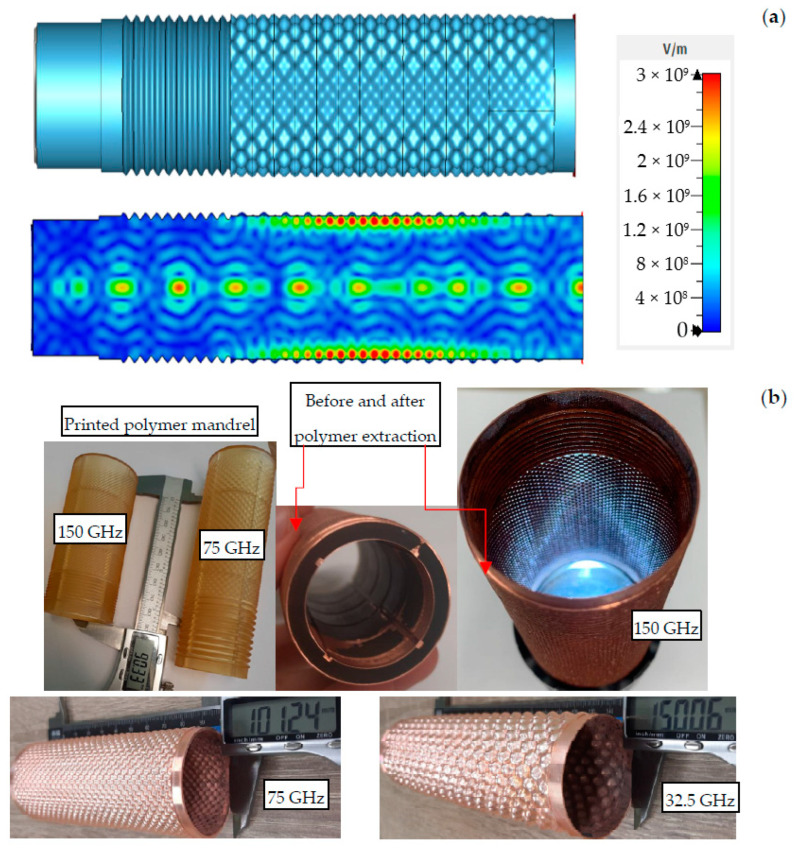
(**a**) Scheme of the electrodynamic system for Cherenkov oscillators consisting of the 2D-periodical SWS and the up-stream Bragg reflector (top) and the results of the 3D CST PIC simulations of the generator in Ka-band (bottom); (**b**) Photographs of the stages of creating an all-metal 2D-periodical SWS for operation in various frequency ranges.

Electrodynamic systems for implemented Cherenkov oscillators are shown in Figure 9a. The profile of the inner surface of the element has a two-dimensional sinusoidal corrugation (the 2D-periodic SWS in the interaction region) and a one-dimensional axially symmetric corrugation (up-stream Bragg reflector). In the developed concept of generators, the use of the 2D-periodic SWS ensures the coupling and mutual scattering of the four partial wave-fluxes: two wave-beams propagating in the forward and backward (relative to the electron beam propagation) axial directions, which are responsible for the effective interaction with the electrons (as in conventional analogues exploiting 1D-periodic SWS), as well as two wave-beams propagating in azimuthal clockwise and counterclockwise directions, which provide synchronization of the radiation of the spatially-extended tubular electron beam. Meanwhile, the upstream Bragg reflector installed on the cathode side provides a reflection of the backward wave-flux and single-directional radiation output from the collector side of the generator.

The corrugation parameters are determined by the operating frequency range. In the experiments, Cherenkov oscillators up to the G-band were elaborated with the following parameters: operating frequency is up to 150 GHz, corrugation period is up to 2 mm, and corrugation depth is up to 1 mm. The oversize factor Ø/λ is about 20. As it was mentioned above, the Bragg resonator was printed for these high-power generators, the outer surface of which was the negative of the inner surface of the original model. The dimensions were measured, and the shrinkage of the polymer was evaluated. After adjusting the model (about a 0.3% increase in the model in all coordinates), a hollow cylinder was printed with the required corrugation on the outside surface. At the same time, grooves were applied on the inner side along and across the entire structure, so that the minimum wall thickness was 1 mm and the maximum was 3 mm. This solution greatly facilitates the process of removing the polymer blank during its further destruction under thermal cycling.

After the successful printing of the electrodynamics systems (6 h), their cleaning and preparation, primary metallization (2 h), and further growth by the galvanic method to a thickness of 1 mm (40 h) were carried out. At the same time, the process was carried out in three parts at once (limited by the size of the print area of the printer and the size of the chemical baths). When the copper surface was ready, the polymer structure was destroyed by periodic exposure to hot water and liquid nitrogen and was easily removed mechanically by hand. As a result, all-copper electrodynamic structures were realized (stages of the creation of these structures are shown in Figure 9b) and successfully tested in high-power experiments [23,24].

#### 3.2.2. High-Power Continuous Wave (CW) Applications without Active Cooling Requirements

**Figure 10:**
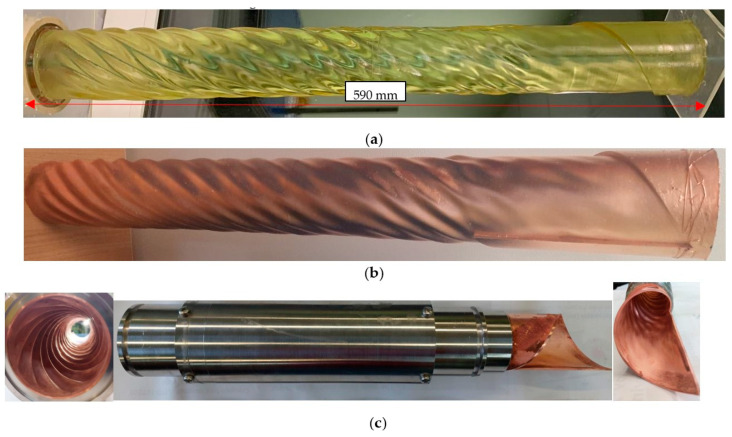
(**a**) Photograph of the converter mandrel; (**b**) copper-plated 3D-printed converter; (**c**) Final view of electrodynamic structure.

Mostly, a Gaussian wave beam is required by a customer as the output structure of a high-power microwave source. However, operating types of electronic device oscillations have different RF-field distributions. Thus, such electrodynamic converters are in great demand. To do this, profiles of the waveguide path are synthesized in a certain way, which have an extremely complex asymmetric shape. Meanwhile, the length of such waveguides can reach tens of centimeters, which makes the task of their creation on CNC machines extremely expensive and difficult. An example of this is a converter from the operating mode of the gyrotron TE_12_ with a waveguide outlet 66 mm in diameter into a Gaussian beam. The length of this electrodynamic element is 590 mm; the surface profile is shown in Figure 10. The initial view of the 3D-printed blank is shown in Figure 10a, with primary metallization in Figure 10b. This technological process took about a day, while chemical copper plating took about a couple of hours. For a more uniform growth of this structure, special cylindrical-type galvanic baths with a closed electrolyte circulation system with filtration from the formed anode sludge (the main source of the formation of large-scale dendrites that make the copper layer loose) were developed. As a result of continuous galvanic copper plating, a copper layer of 2.5 mm was grown, and the mandrel was successfully extracted (Figure 10c).

**Figure 11:**
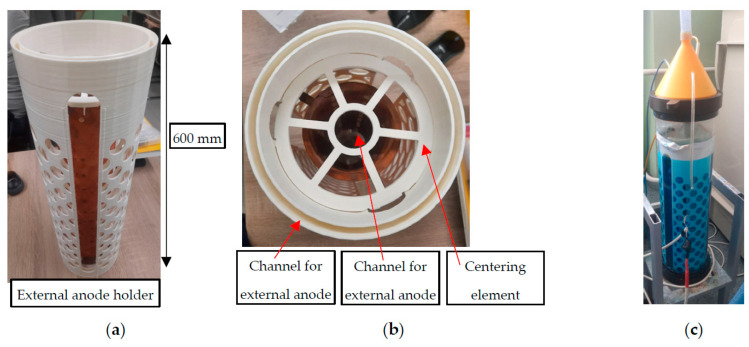
Photo of the galvanic system: (**a**) external anode holder, side view; (**b**) view from above; (**c**) assembled appearance.

To provide uniform, continuous, long-term growth, a special galvanic bath was developed in the form of a cylindrical flask. It was assumed that the item should be coated and built up vertically to avoid the possibility of forming bends over a long length of polymer. To set copper anodes, a special part was printed, which was installed along the entire length along the edges of the bath (Figure 11a). To center the coated element relative to the anodes, auxiliary elements were also printed, which were attached to the anode holder (Figure 11b). As for the additional possibility of building up the inner surface, the design of the centering elements involves the installation of anodes and their centering inside the printed element. A photo of this galvanic system is shown in Figure 11c. To provide electrolyte purification, this electroplating bath was assembled as a closed system with electrolyte circulation through a filtration system. The supply of electric current through the wires into the system was carried out through special holes in the lid of the bath, which were closed by hot glue during operation.

The growth of the copper layer in the region of the axially asymmetrical cut was controlled by a specially selected shape of the polymer blank. The boundary, beyond which copper should not grow, is made in the form of a “curb”. It has a significantly larger transverse dimension. This area is easy to cover with a varnish that prevents chemical copper plating of the surface. It is also even easier to machine this part at the radius of the “curb”, while the desired layer of copper will not be touched due to its location on smaller transverse dimensions.

Having received a copper-plated element, similarly to the case with the Bragg resonators described above, the polymer mandrel was removed. The key difference from the previous case was the significantly larger dimensions of the part, as well as the provision of directed growth of the copper layer and the creation of an axially asymmetric shape of the copper element. The measurements of the geometric dimensions of the final part showed full compliance with the design documentation, which meant the successful completion of the task.

#### 3.2.3. High-Power CW Applications with Power Load and Active Cooling Demands

**Figure 12:**
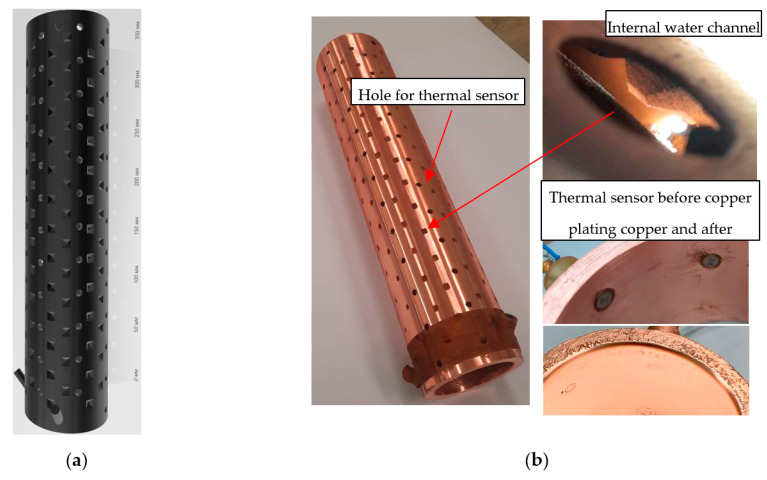
(**a**) The structure of the collector model and its printed version; (**b**) The final view of the collector after copper plating and removal of the polymer mandrel.

A separate area of CMPS technology for creating heat-loaded elements is structures with complex geometry that require active liquid cooling and, therefore, closed channels for transporting the coolant. The key difference from the previous case is the impossibility of mechanical removal of the photopolymer mandrel. For instance, consider an example of creating a collector of a powerful (55 kW in a beam) technological gyrotron [25], on the basis of which the technology was optimized. The structure of the collector model is shown in Figure 12a. It is complicated by the presence in the water channel of special teeth for turbulent fluid flow, as well as temperature sensors integrated into the body of copper. In this case, the inner wall of the collector must withstand an electron beam deposition power density of up to 600 W/cm^2^.

The required smooth inner and outer surfaces of the collector, as well as a fairly large inner diameter, allow further simple processing on a lathe. This means that the galvanic build-up of a thick layer can be carried out on all sides with further removal of inaccuracy from the machine. As in the previous case, a blank was printed with an inverse shape, taking into account both thermal sensors and geometry for a turbulent fluid flow (see Figure 12a). However, building up a thick layer of copper on all sides of the model does not imply further mechanical extraction of the polymer mandrel. In this case, the process of removing the polymer occurs by means of melting. For this, a special composite polymer was used. A large concentration of one of its components at temperatures above 300 °C evaporates without a trace. Another substance that holds the shape of the 3D-printed structure forms carbon deposits on the inner copper walls at temperatures above 500 °C. The technological process of removing the coal layer involves heat treatment at more than 850 °C. Under such conditions, the structure of the layer ceases to be unified, crumbling into loose ashes. However, such extreme temperatures for copper elements with a complex surface shape, often having rather thin-walled sections, may not withstand mechanical stresses and change their shape. In general, processing takes place at temperatures up to 600 °C, when the main part of the polymer flows out of the copper-plated product at a temperature of about 300 °C. In the case of the correct execution of the heating and cooling stages, most of the coal-thin shell (about 100 microns) on the copper walls easily remains at the slightest load. For example, this process is facilitated by holding the element in an ultrasonic bath and further flushing the channel under pressure. The oxide and residues of combustion processes on the copper surface are subsequently removed by chemical cleaning, for example, in hydrochloric or nitric acid.

In this way, several collectors for the research on technological gyrotrons were created, as shown in Figure 12b. The thickness of the copper layer of the external wall was 3 mm, and that of the inner wall was 1.5 mm. After heat treatment and removal of the polymer, the outer part was turned on a conventional lathe. This was necessary for the further installation of copper-plated polymer sleeves with sensors. The polymer sleeve prevents effective cooling of the sensor, the side surface of which is located in the water channel of the collector. The copper-plated end of the sensor, without a polymer layer, rises with the inner surface of the collector and is overgrown with copper, about 1 mm thick. Thus, a single vacuum-tight copper layer is created on the inner surface. At the same time, the high thermal conductivity and sensitivity of the sensor make it possible to effectively evaluate the distribution of the thermal load at a given point.

## 4. Discussion

In the process of testing, adapting, and optimizing the CMPS technology, a wide range of applications in the field of microwave electronics were investigated. In some areas, the created components have passed a full cycle of tests, showing their successful further application [23,24] and development paths (in particular, Section 3.2.1). Despite the successful application of the technology in the high-frequency range [19,20] (Section 3.1), the development of the technology requires further active participation in the creation of prototypes and their testing in view of the large number of nuances related to the surface geometry, alignment of parts of elements, error control, etc. Advancement into the higher frequency range (THz and above) [15,26] appears to be limited at the moment by the accuracy of 3D printing. However, this industry is rapidly evolving; the resolution of SLA printer matrices is increasing [17], and more complex MJP printing technologies [27] are also improving their characteristics. More exotic 3D printing technologies with nanoscale parameters are emerging [28], which can also be used in the future as blanks for further metallization. In the field of creating large-scale elements (Section 3.2.3), there is great interest in further development in the form of a combination of small-scale and large-scale elements in order to manufacture a single device [12] (for example, combining a gyrotron cavity, a converter, a transmission line, and an electron optics element).

## 5. Conclusions

A detailed analysis of the application of CMPS technology in relation to the manufacture of various components of microwave electronics has been carried out. The processes for creating a high-quality copper layer on the surface of 3D-printed polymer parts with complex surface shapes have been optimized. The roughness of the resulting 3D-printed surface, the effects of shrinkage on the material, and its compensation were studied to minimize the inaccuracy of the created elements. The printing process has been optimized in terms of providing a smoother surface as well as further removal of the polymer blank from the copper-plated part.

Thus, key components of electronic devices for their class have been successfully created and experimentally tested, namely:

- For measurements at low power levels (on vector network analyzers), the technology for creating electrodynamic components at frequencies up to 700 GHz has been developed and successfully tested in experiments. These products have a thin copper layer (10–50 microns, significantly greater than the depth of the skin layer), which makes the process of their creation extremely cheap and fast, which is especially important in cases of test experiments on the performance of theoretical ideas.
- Significant scientific results were obtained in the process of testing the created all-metal resonators with complex internal surface corrugation under conditions of super-power pulsed RF fields (up to ~200–250 MW power level in the Ka-band and ~120–150 MW in the W-band) and electron beams (up to 2.5 GW).
- A process has been implemented to create large-sized (more than 600 mm in length) all-copper waveguide elements with a complex internal surface structure, tested under conditions of continuous operation with a radiation power of several tens of kW at a frequency of 28 GHz.
- The procedure for creating an all-copper, thick-walled copper electro-optical component with internal cavities that act as cooling channels has been successfully completed. The product is made in the form of a single part, thanks to the use of a burnable polymer and a specially shaped mandrel model.

The experimental data obtained in each direction of the research indicate the potential possibility of an integrated approach to the creation of high-frequency electronic devices using additive methods, creating both electron-optical and electrodynamic units. New opportunities appear in order to create inexpensive resonators with complex shapes that provide highly selective properties. In the process of synthesizing irregular waveguides, it is possible to significantly reduce the restrictions on the complexity of the surface profile, which will allow for more compact and efficient line-mode converters. Installing sensors inside the manufactured element while maintaining operability under thermal load under high vacuum conditions will allow more detailed studies of expensive vacuum devices, i.e., the influence of microwave radiation generation on the topology of the power density profile of the electron beam on the collector, the influence of secondary emission, etc. The authors of the article are ready for active cooperation in the field of testing the developed technology in various areas of scientific and industrial fields.

## Figures and Tables

**Table 1:** Main characteristics of the resins used.

Parameters	Gorky Liquid Force	Gorky Liquid Castable
Shore hardness (D scale)	77	72
Charpy impact strength without notch (kJ/m^2^)	41.30	
Notched Charpy impact strength (kJ/m^2^)	3.88	
Tensile Strength (MPa)	46.80	11.58
Elongation at break (%)	19	
Flexural modulus (MPa)	1366.00	
Bending stress (MPa)	56.50	33.6
Density at 20 °C, kg/m^3^		1160
Dynamic viscosity (mPa×s) 23 °C		110
Coefficient of thermal expansion (μm/m/°C)	80	
Exposure time (s), Phrozen 8K Mega (100 μm Z-step)	9.1	11.2
Exposure time (s), Phrozen 8K Mini (100 μm Z-step)	7.8	7.5

## Data Availability

Not applicable.
